# Chemicals from ethanol: the acetone synthesis from ethanol employing Ce_0.75_Zr_0.25_O_2_, ZrO_2_ and Cu/ZnO/Al_2_O_3_

**DOI:** 10.1186/s13065-017-0249-5

**Published:** 2017-04-04

**Authors:** Clarissa Perdomo Rodrigues, Priscila da Costa Zonetti, Lucia Gorenstin Appel

**Affiliations:** Divisão de Catálise e Processos Químicos, Instituto Nacional de Tecnologia, Av. Venezuela 82/518, Saúde, Rio de Janeiro, RJ CEP 21081-312 Brazil

**Keywords:** Acetone, Ethanol, Zirconia, Copper, Ceria, Acetaldehyde, Acetate, Cu/ZnO/Al_2_O_3_

## Abstract

Acetone is an important solvent and widely used in the synthesis of drugs and polymers. Currently, acetone is mainly generated by the Cumene Process, which employs benzene and propylene as fossil raw materials. Phenol is a co-product of this synthesis. However, this ketone can be generated from ethanol (a renewable feedstock) in one-step. The aim of this work is to describe the influence of physical–chemical properties of three different catalysts on each step of this reaction. Furthermore, contribute to improve the description of the mechanism of this synthesis. The acetone synthesis from ethanol was studied employing Cu/ZnO/Al_2_O_3_, Ce_0.75_Zr_0.25_O_2_ and ZrO_2_. It was verified that the acidity of the catalysts needs fine-tuning in order to promote the oxygenate species adsorption and avoid the dehydration of ethanol. The higher the reducibility and the H_2_O dissociation activity of the catalysts are, the higher the selectivity to acetone is. In relation to the oxides, these properties are associated with the presence of O vacancies. The H_2_ generation, which occurs during the TPSR, indicates the redox character of this synthesis. The main steps of the acetone synthesis from ethanol are the generation of acetaldehyde, the oxidation of this aldehyde to acetate species (which reduces the catalyst), the H_2_O dissociation, the oxidation of the catalyst producing H_2_, and, finally, the ketonization reaction. These pieces of information will support the development of active catalysts for not only the acetone synthesis from ethanol, but also the isobutene and propylene syntheses in which this ketone is an intermediate.

**Electronic supplementary material:**

The online version of this article (doi:10.1186/s13065-017-0249-5) contains supplementary material, which is available to authorized users.

**Graphical abstract:**
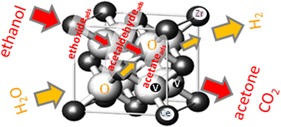
Acetone from ethanol.

## Background

Acetone is an important solvent and widely used in the synthesis of drugs and polymers. The most significant industrial application of this ketone is the manufacture of the precursor for the synthesis of methyl methacrylate and meta-acrylic acid, which are monomers of polymers highly demanded nowadays. It is also employed in the synthesis of bisphenol-A (BPA polycarbonate), methyl isobutyl ketone and isopropanol among others [1]. Currently, acetone is mainly generated by the Cumene Process, which employs benzene and propylene as fossil raw materials. Phenol is a co-product of this synthesis [2].

At presents, ethanol is considered a special platform molecule [3, 4] since it can produce many chemicals employing one-pot processes and multifunctional catalysts. Acetone [5], ethyl acetate [6–9], *n*-butanol [10, 11], acetic acid [12], propylene [13], isobutene [14] and 1,3-butadiene [15] are good examples of these syntheses.

The acetone synthesis from ethanol is quite interesting because not only this alcohol is a renewable feedstock, but also it does not generate phenol as a by-product.

Recently, both Iwamoto [13] and Liu et al. [14] have suggested that acetone is the intermediate of the propylene and isobutene syntheses from ethanol, respectively. Surely, the understanding of the acetone synthesis will support future developments related to these subjects.

Reaction 1 shows the synthesis of acetone from ethanol:

1 $$2 {\text{ C}}_{ 2} {\text{H}}_{ 5} {\text{OH }} + {\text{ H}}_{ 2} {\text{O}} \to {\text{CH}}_{ 3} {\text{COCH}}_{ 3} + {\text{ CO}}_{ 2} + {\text{ 4H}}_{ 2}$$

Many different catalytic compositions have been employed for this synthesis. Murthy et al. [16] studied catalytic systems based on Fe, i.e., Fe_2_O_3_–ZnO, Fe_2_O_3_–CaO and Fe_2_O_3_–Mn. Nakajima et al. [17–19] worked with mixed oxides based on Fe–Zn and Zn–Ca, which showed high activities and selectivities to acetone. Furthermore, Nishiguchi et al. [20] investigated the ethanol reforming reaction and noticed that acetone is a by-product when employing Cu/CeO_2_. Bussi et al. [21] verified that Cu/La_2_Zr_2_O_7_ produced high yields of acetone. Finally, Idris and Seebauer [22] and Yee et al. [23] studying ethanol reactions observed that Pd/CeO_2_ and CeO_2_ also synthesize acetone.

As envisioned, some physical–chemical properties of the catalysts might be relevant for the acetone synthesis. However, there are very few correlations between the properties mentioned above and their catalytic performance.

Moreover, despite the pieces of information available the reaction steps related to the acetone synthesis from ethanol have not been well established yet.

Rodrigues et al. [5], using physicals mixtures composed of Cu/ZnO/Al_2_O_3_ + ZrO_2_ and other oxides, IR spectroscopy and catalytic tests at different experimental conditions, proposed the following reaction system. Firstly, ethanol is dehydrogenated to acetaldehyde on Cu surface; secondly, it migrates to the oxide surface and is oxidized to acetate (carboxylate species); finally, these species condensate and generate acetone and CO_2_. In order to regenerate the oxide surface (Mars and Van Krevelen mechanism, [24]), it was suggested that H_2_O should be dissociated on the Cu surface generating oxidant species which then migrate to the oxide and regenerate its surface. This proposal is based on the works of Idris & Seebauer [22], Yee et al. [23], and also on the research carried out by Voss et al. [25], which is related to the oxidation of ethanol employing H_2_O as an oxidant agent.

Recently, Iwamoto [13] studying the propene generation from ethanol proposed two different mechanisms for the acetone synthesis. This author suggested that when Sc/In_2_O_3_ is employed as a catalyst, ethanol generates acetaldehyde, which is then oxidized by H_2_O or surface hydroxyl groups to acetic acid. After that this compound reacts producing acetone and CO_2_ by the ketonization reaction. When the catalyst is Y_2_O_3_–CeO_2_, acetaldehyde is converted to ethyl acetate and then this ester decomposes to form acetic acid and ethene. Finally, this acid synthesizes acetone and CO_2_ by the ketonization reaction.

Rodrigues et al. [5] analyzed the acidity and basicity of physical mixtures composed of Cu/ZnO/Al_2_O_3_ and oxides. However, due to the adsorption of acetaldehyde generated by Cu/ZnO/Al_2_O_3_ on the oxides (described below), it was not possible to fully describe the role of these properties. Moreover, the proposed mechanism suggests oxi-reduction steps and employs H_2_O as an oxidant agent. Thus, it will be relevant to analyze the role of the H_2_O dissociation step and redox properties of the catalysts in the acetone synthesis.

As a result, the aim of this work is twofold: firstly, show the influence that the physical–chemical properties of three different catalysts have on the acetone synthesis steps and, secondly, employing these pieces of information together with the data generated by the TPSR followed by IR-MS improve and confirm the mechanism proposed by Rodrigues et al.

## Results and discussion

Three catalysts were employed in this work. Two of them commercial catalysts, (CuO/ZnO/Al_2_O_3_, CZA) and monoclinic ZrO_2_), as described in the methodology, and a third one was synthesized in the laboratory (Ce_0.75_Zr_0.25_O_2_, CeZr).

The CeZr catalyst was analyzed by Raman spectroscopy and XRD, which confirmed that the mixed oxide was synthesized, i.e., Zr is in the lattice of CeO_2_ [26].

**Fig. 1:**
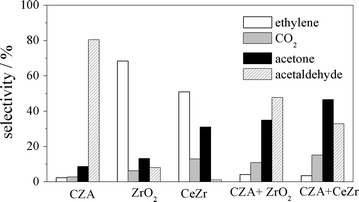
Selectivities to acetone, ethene, carbon dioxide, acetaldehyde of CZA, CeZr, ZrO_2_ and CZA + CeZr, CZA + ZrO_2_ at ethanol isoconversion (~ 35%)

Figure 1 shows the selectivity to acetone, acetaldehyde, ethylene and CO_2_ at isoconversion (~35%), employing CZA, ZrO_2_, CeZr, CZA + ZrO_2_ (1:1) and CZA + CeZr (1:1). Table 1 depicts the generation rates of the same compounds using CZA, ZrO_2_ and CeZr. It also exhibits the ratio between the oxygenate compounds syntheses and ethanol total consumption rates (R_oxyg_), the ratio between the ethylene formation and ethanol total consumption rates (R_olef_), and, the ratio between the acetone generation and the oxygenate compounds syntheses rates (R_acet_). The catalytic tests produced H_2_ and very small amounts of methane, CO and propylene as well. The ethanol conversions of ZrO_2_ and CeZr are stable during 12 h (time on stream), whereas CZA exhibits a decrease of the conversion in the first 2 h and after that it remain stable (~10 h), (see Additional file 1: Figure S1). Thus, the deactivation phenomenon is not relevant at these experimental conditions. The results presented below were analyzed considering the acetone synthesis steps described in the background.

**Table 1:** Kinetic data

Samples	r_ace_	r_act_	${\rm{r}}_{\rm{co}_{2}}$	r_et_	R_oxyg_	R_olef_	R_acet_
ZrO_2_	5.4	4.9	5.7	18.6	0.46	0.54	0.34
CeZr	15.0	12.8	18.2	3.1	0.94	0.06	0.33
CZA	0.0	619.4	3.1	0.0			

Acetone (r_ace_), acetaldehyde (r_act_), ethylene (r_et_) and CO_2_ (r_CO2_) formation rates (µmolg_cat_^−1^ min^−1^). Ratio between the rates of ethanol consumption for oxygenate syntheses and ethanol total consumption (R_oxyg_), ratio between the rates of ethylene formation and ethanol total consumption (R_olef_) and ratios values between the acetone generation rate and the oxygenated formation rate (R_acet_)

### The dehydrogenation versus the dehydration of ethanol

Taking R_oxyg_ and R_olef_ values (Table 1) into account and employing ZrO_2_ as catalyst, it can be said that when 1 mol of ethanol reacts 54% is transformed into ethylene and the remaining 46% into oxygenated compounds. When using CeZr, it can also be inferred that, at the same conditions, 6% of the mols of ethanol are transformed into ethylene and 94% into oxygenated compounds. On the one hand, ZrO_2_ is more active than CeZr for the dehydration of ethanol. On the other hand, CeZr is much more active for the syntheses of the oxygenate compounds when compared with ZrO_2_.

The first step of the acetone synthesis is the acetaldehyde generation. As can be observed (Table 1), CZA shows high rate of acetaldehyde generation. Considering that Cu is the main component of this catalyst, it can be suggested that this aldehyde is generated by the dehydrogenation of ethanol on the Cu^o^ surface [8]. This catalyst shows very low selectivity to ethylene.

The oxides, i.e., CeZr and ZrO_2_ generate acetaldehyde (Table 1). They also produce acetone and CO_2_ from this aldehyde. Di Cosimo et al. [27] described the mechanism of the acetaldehyde synthesis on oxides according to the following steps: firstly, the H of the OH of ethanol is abstracted by a strong basic site generating ethoxide species, which are then adsorbed on acid sites. The generation of ethoxide species occurs on a pair of sites being one acid and the other one a strong base. Then, α-H is abstracted from the ethoxide species by another strong basic site and acetaldehyde is obtained. The ZrO_2_ and CeZr oxides show acid and strong basic sites (Table 2). Thus, the acetaldehyde generation of these oxides can be associated with their acidity and basicity.

**Table 2:** Physical-chemical properties of the catalysts

Samples	S m^2^g^−1^	D_B_ μmolg^−1^			D_A_ u.a.g^−1^	δ_A_ cm^−1^
		W	M	S		
*m*-ZrO_2_	110	73	92	99	184	20
CeZr	123	43	71	103	60	14
CZA	114	14	8	–	~0	–

Specific surface area (S), densities of basic sites (D_B_, weak, W; medium, M; and strong, S), optical densities of the band at 1445 cm^−1^ obtained by pyridine adsorption at 25 °C (D_A_) and the shift values of the 8a mode (δ_A_)

Moreover, the acetone synthesis occurs in a redox environment (see “Background” section). Therefore, the acetaldehyde synthesis by the oxidative dehydrogenation of ethanol cannot be ruled out neither for CZA nor the oxides [28].

Table 1 depicts that CeZr and ZrO_2_ generate ethylene. This olefin is obtained by the dehydration of ethanol. Considering that CeZr and ZrO_2_ do not show Brönsted acid sites, two mechanisms can be proposed: E_1cB_ and E2. Both are associated with pairs of Lewis acid and basic sites [27]. The former mechanism is related to strong basic and weak acid sites. Initially, ethoxide species are generated. After that another basic site abstracts a β-H of the ethoxide producing ethylene. The E2 mechanism is related to the simultaneous abstraction of OH and β-H by a pair of acid and basic sites. According to Parrot et al. [29], for the E2 mechanism, the higher the strength of the acid sites, the lower the activation energy for the β-H abstraction is, and consequently, a higher rate of ethylene synthesis is observed.

Table 1 depicts that ZrO_2_ shows a higher ethylene generation rate than CeZr. Comparing ZrO_2_ with CeZr (Table 2), it can be inferred that the former exhibits not only a higher density of acids sites, but also stronger acid sites than those of CeZr, as indicates the upward shift of the 8a vibration ring of the piridine adsorption (δ_A_) [30], whereas both oxides show the same density of strong basic sites. It can be suggested that it is the acidity which controls this catalytic behaviour. These oxides may follow the E2 mechanism.

As is well known, the acetaldehyde generation is the first step of the acetone synthesis. Thus, the selectivity to acetone is associated with the competition between the dehydration and dehydrogenation of ethanol. In the case of CeZr and ZrO_2_, Tables 1 and 2 show that the higher the acidity of the catalyst, the lower the rates of acetone, CO_2_ and acetaldehyde synthesis are. Thus, the acid and basic properties of the catalysts are relevant properties for the synthesis of this ketone from ethanol.

In the case of CZA, as almost only acetaldehyde is observed, the dehydration versus dehydrogenation competition seems not to be relevant.

### The redox step

#### The redox step and the oxides (CeZr and ZrO_2_)

The second step of the acetone synthesis is related to the oxidation of acetaldehyde to acetate species (the redox step) [5].

Figure 1 shows that adding ZrO_2_ or CeZr to CZA (physical mixtures) once again acetone, CO_2_, acetaldehyde and ethylene are generated. It is interesting to observe that the physical mixtures show very low selectivity to ethylene and high selectivities to acetone when compared with the oxides.

According to the mechanism proposed by Rodrigues et al. [5], acetaldehyde is mainly generated on CZA and then it migrates to the oxide. The adsorption of acetaldehyde on the acid sites of these oxides hinders the dehydration reaction. This adsorption might be the reason for the low selectivity to ethylene and high towards acetone as observed in the case of the physical mixtures when compared to CeZr and ZrO_2_.

Thus, using physical mixtures it is possible to compare the catalytic behavior of ZrO_2_ and CeZr without the interference of their acid properties.

On the one hand, the physical mixture comprising of CZA + CeZr generates higher selectivity to acetone and CO_2_ than the one composed of ZrO_2_. On the other hand, CZA + ZrO_2_ shows higher selectivity to acetaldehyde than the one comprised of CeZr (Fig. 1).

Considering that acetaldehyde is mainly generated on CZA and these oxides might show the same behavior for the ketonization reaction (same density of strong basic sites, see the next topic), it can be suggested that the catalytic behavior of these physical mixtures is associated with the acetaldehyde oxidation rate (the redox step) [5]. This rate seems to be slower for ZrO_2_ when compared with CeZr.

The WGS reaction (water gas shift reaction) and the redox step of the acetone synthesis are very similar. Both refer to the oxidation of CO to COO species employing O species generated by the H_2_O dissociation.

Since the 90s, the WGS reaction mechanism employing Pt and other metals supported on reducible oxides has been subject to discussion. Nowadays, the Redox mechanism seems to prevail [24, 31–34]. The WGS Redox mechanism refers to the Mars Van Krevelen mechanism [24]. In short, firstly, the O (oxide) of interface metal-reducible oxidizes CO to CO_2_. After that, H_2_O is dissociated on the interface and reoxidizes the oxide.

**Fig. 2:**
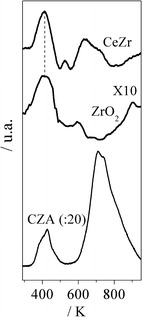
TPD-H_2_O over CeZr, ZrO_2_ and CZA

According to Rodrigues et al. [5] H_2_O is dissociated on Cu^o^ (CZA) during the acetone synthesis when employing CZA + ZrO_2_. The oxidant species generated from this dissociation might spillover toward the oxide and then reoxidize it (reduced by acetaldehyde). Figure 2 exhibits the TPD-H_2_O of CeZr, ZrO_2_ and CZA. It can be observed that CZA generated a large peak of H_2_. Thus, this catalyst is very active for the H_2_O dissociation when compared with CeZr or ZrO_2_.

In the case of the physical mixtures, the oxidant agent is mainly supplied by the dissociation of H_2_O on Cu^o^, which occurs equally for both mixtures. Therefore, the catalytic performance of the physical mixtures is related to the acetaldehyde oxidation or, in other words, to the reduction of the oxides.

**Fig. 3:**
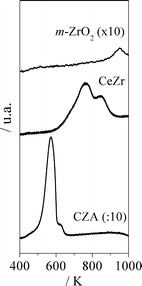
TPR profiles of ZrO_2_, CeZr and CZA

Figure 3 exhibits the TPR profiles of the catalysts. The mixed oxide shows a broad peak with a maximum at 489 K and a shoulder at 573 K, whereas only a tiny peak is noticed for ZrO_2_. It can be deduced that the reducibility of CeZr is much higher than the one of ZrO_2_. Therefore, the CZA + CeZr and CeZr higher selectivities to acetone can be associated with the reducibility of CeZr.

Recently, Zonetti et al. [31] studying the RWGS reaction showed that the WGS Redox mechanism can also be considered for some oxides or mixed oxides without the presence of metal. Thus, the dissociation of H_2_O on the oxides is also relevant (see Fig. 2).

Chen et al. [35] studied the dissociation of H_2_O on CeO_2_. They showed that the adsorption of H_2_O on this oxide (reduced) creates hydroxyls species, which then react desorbing H_2_O and generating O_lattice_ and O_vacancy_. These hydroxyl species also react, producing H_2_ and O_lattice_, thus reoxidizing CeO_2_. The authors demonstrated that the presence of O vacancies on the CeO_2_ surface promote the formation of H_2_. In other words, the O vacancies on CeO_2_ control the reactivity of the surface hydroxyls species.

As it is well known, CeZr and ZrO_2_ show superficial vacancies. Considering the similarity between the redox step and the WGS reaction, it can be proposed that acetaldehyde, which is synthesized by these oxides according to the mechanism discussed above, is oxidized to carboxylate species by the O species of the lattice of these oxides, thus reducing them. After that, H_2_O is dissociated on the superficial vacancies of these reduced oxides. Then CeZr and ZrO_2_ are oxidized and H_2_ is desorbed. This mechanism suggests that not only the reducibility, but also the activity towards the H_2_O dissociation of the catalysts, are very important properties for the redox step of the acetone synthesis.

Figure 2 depicts the TPD of H_2_O on the CeZr and ZrO_2_ reduced samples. It was verified (not shown) that the molecular desorption of H_2_O occurs from low to high temperatures, which is related to the recombination of hydroxyls species [35]. The H_2_ spectra show that both oxides generate this gas at low temperature (413 K). However, the mixed oxide produces a much higher amount of H_2_ than ZrO_2_ (Fig. 2).

These results (Figs. 1, 2, 3) suggest that CeZr is more active for the acetone generation compared with ZrO_2_ not only due to its acid properties, but also its reducibility and reactivity toward the H_2_O dissociation.

#### The redox step and CZA

Figure 3 shows the TPR profile of CZA. It can be verified that CuO, the main component of this catalyst, is reduced at low temperature.

According to Phatak et al. [36], Fig. 2 exhibits that CZA produces a large amount of H_2_. It is interesting to verify that the first peak of H_2_ occurs at very low temperature.

Therefore, it can be proposed that the decomposition of H_2_O oxidizes Cu^o^ to CuO. After that, the oxidation of acetaldehyde to acetate species might occur on this oxide according to the Mars and Van Krevelen mechanism [24]. However, the Langmuir–Hinshelwood mechanism, as proposed by Voss et al. [25], cannot be ruled out.

When considering the H_2_ spectra of ZrO_2_ (Fig. 2) and its catalytic behavior compared with the one of its physical mixture, it can be suggested that the dissociation of H_2_O on CZA generates O species which show mobility and promote the redox behavior of ZrO_2_, as proposed by Rodrigues et al. [5]. These results support the Langmuir–Hinshelwood mechanism for the H_2_O dissociation on CZA.

Indeed, the copper-based catalyst (CZA) shows a very low selectivity to acetone (Fig. 1) and a high one to acetaldehyde. It can be suggested that this aldehyde desorption rate is higher than the one of its oxidation in spite of the CZA properties.

### The acetone generation step

#### The ketonization step and the oxides (CeZr and ZrO_2_)

The next and last step of the acetone synthesis is the condensation of acetate species, which generate acetone and CO_2_ (ketonization reaction). Strong basic sites promote this condensation reaction [37, 38]. Table 2 depicts that ZrO_2_ and CeZr almost show the same density of strong basic sites. Thus, for this step, the oxides might show similar behavior.

#### The ketonization step and CZA

Table 2 depicts that the Cu based-catalyst show only weak and medium strength basic sites. It is a well-known that strong basic sites promote the ketonization step [37, 38]. Thus, the rate of the acetate condensation might be affected by the basicity of CZA.

When CZA is employed, Table 1 and Fig. 1 exhibit that almost only acetaldehyde is generated. However, it is worth mentioning that this catalyst, at high residence time (500 mg, 60 mL min^−1^, 673 K), shows high selectivity to acetone (50%) at high conversion. The selectivities to acetaldehyde, CO_2_ and propene are 25, 10 and 10%, respectively. These results show that CZA is able to synthesize acetone. However, it is less active than the oxides.

The Cu based catalyst almost does not show acid sites. As well known, acetaldehyde is kept on the surface by the acid sites. The low concentration of these species might contribute to the acetaldehyde desorption. At high residence time (see above), the readsorption of acetaldehyde might enhance the selectivity to acetone.

#### The TPSR followed by IR-MS (DRIFTS) spectroscopies

Aiming at better describing the synthesis of acetone from ethanol, CeZr and CZA were analyzed by TPSR (temperature programmed surface reaction*)* of ethanol followed by IR and MS online analyses. Taking into account the ZrO_2_ similar catalytic behavior when compared with CeZr, the TPSR experiments were not carried out for this oxide.

**Fig. 4:**
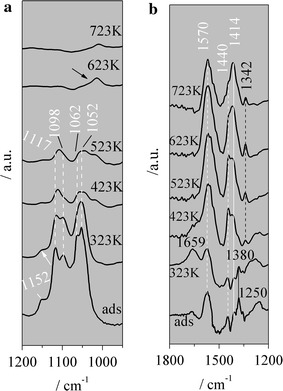
**a** TPSR, IR spectra of CeZr. **b** TPSR, IR spectra of CeZr

Figure 4 shows the IR spectra of the TPSR of ethanol/H_2_O on CeZr. The following absorptions are observed at the ethanol adsorption and also at low temperature in the range of 1200–950 cm^−1^: 1152, 1117, 1098, 1062, 1052 cm^−1^ (Fig. 4a). These bands can be associated with the ethanol dissociative adsorption, which generates ethoxide species. Figure 4a exhibits that as the temperature increases the intensities of these absorptions decrease. According to Finocchio et al. [39], the absorption at 1152 cm^−1^ can be associated with ethoxide species adsorbed on Zr^+4^. They also observed the on-top and doubly methoxide species adsorbed on Ce^+4^ ions. Binet and Daturi [40] verified that the reduction of CeO_2_ and Ce_x_Zr_1–x_O_2_ down shifts the on-top ethoxide absorptions, whereas the doubly bridging ones move to higher frequencies. Thus, the vibrations at 1117 and 1052 cm^−1^ can be assigned to on-top and doubly bridging vibrations associated with ethoxide species adsorbed on Ce^+4^, respectively, whereas 1098 and 1062 cm^−1^ to on-top and doubly bridging vibrations of ethoxide species adsorbed on Ce^+3^, respectively. As a result, ethoxide vibrations show that the CeZr surface is partially reduced at the ethanol adsorption, and also at higher temperatures.

Figure 4b depicts the TPSR spectra at the 1800–1200 cm^−1^ range. The vibrations at 1570, 1440 and 1414 cm^−1^ observed from the adsorption of ethanol until high temperatures can be assigned to ν_as_(OCO), δ_as_(CH_3_), ν_s_(OCO) of the acetate species, respectively [23] (white lines and numbers). The band at 1380 cm^−1^ (δ_s_(CH_3_)) is also related to the ethoxide species (black line) [23].

Given that the catalyst was previously treated with H_2_ at 723 K (see “Experimental” section) and the simultaneous presence of Ce^+4^ and Ce^+3^ on the catalyst surface observed at the ethanol adsorption (IR spectra), it can be inferred that CeZr was not completely reduced. Indeed, this result is in line with the TPR profile of the catalyst mentioned above. Moreover, the acetate species observed at the ethanol adsorption (ads, Fig. 4b) show that the O of the CeZr surface is able to oxidize ethoxide species even at low temperatures reducing the oxide.

Comparing Fig. 4b with 4b (spectra at 323, 423, 523 K) it can be verified that as the temperature increases, the intensities of the ethoxide species vibrations decrease, whereas the ones of the acetate species increase. The O of the CeZr surface oxidizes ethoxide species to acetate species during the TPSR. After that H_2_O might reoxidize the oxide surface. As a result, Ce^+3^ and Ce^+4^ species are on the CeZr surface during TPSR (Fig. 4a, spectra at 323, 423, 523 K).

As the temperature rises the relative intensities of the 1570, 1440 and 1414 cm^−1^ absorptions change. Figure 4b exhibits that the absorptions intensities ratio between 1414 and 1570 cm^−1^ increases as the temperature goes up suggesting that there are carbonates on the CeZr surface (Fig. 4b, spectra at 523, 623 K). This occurs due to the interaction between CeZr with CO_2_. This gas is synthesized by the oxidation of acetate species and/or the ketonization reaction.

According to Yee et al. [23], acetate species are transformed in carbonate species during the TPD of CeO_2_. When it is previously oxidized these authors assigned vibrations at 1568, 1341 cm^−1^ to bidentate carbonate and at 1428 cm^−1^ to symmetric carbonate. When CeO_2_ is previously reduced the same species were observed, i.e., at 1438 (symmetric carbonate), 1538 and 1345 cm^−1^ (bidentate carbonate).

Analyzing Fig. 4b and considering that CeZr might be partially reduced during the TPSR of ethanol, it can be suggested that above 423 K, symmetric and bidentate carbonates are on the reduced and oxidized surface of CeZr. The small absorption at 1013 cm^−1^ (Fig. 4a) can be also associated with carbonate species (arrow). According to Yee et al. [23] the bands at 1438 or 1428 cm^−1^ are the most intense of the carbonate species, which is in line with the changes of the relative intensities of the acetate absorptions described above.

At low temperatures, it is possible to observe a peak at 1659 cm^−1^ and a small shoulder around 1169 cm^−1^, which might be both assigned to acetone adsorbed [41]. As the temperature increases the intensity of this peak (1659 cm^−1^) decreases suggesting that this ketone is desorbed. Moreover, at low temperature a band at 1250 cm^−1^ can be assigned to ethanol adsorbed.

The adsorption of ethanol consumed the OH species. Adding H_2_O, a very broad band at around 3500 cm^−1^ is observed exhibiting the hydroxylation of the catalytic surface during the TPSR (spectrum not shown).

Due to the very high concentration of Cu, low intensity spectra (DRIFTS) were collected for CZA (not shown). However, ethoxide species adsorption at low temperature (323 K) and acetate species at higher temperatures were observed suggesting that the same reaction steps proposed for the oxides might occur in the case of CZA.

**Fig. 5:**
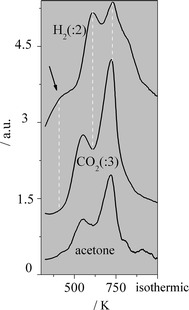
TPSR, MS spectra of CeZr

Figure 5 depicts the spectra of the TPSR-MS (ethanol/H_2_O) on the CeZr catalyst. During this experiment, it was not possible to observe ethanol and acetaldehyde signals. Thus, it can be inferred that these species are oxidized very fast. The acetone and CO_2_ spectra are very similar confirming that both compounds are generated by the same reaction (ketonization). They show two peaks, one at 547 K and another at 720 K.

The H_2_ desorption spectrum shows one shoulder at 412 K (see arrow) and two peaks, one at 610 K and other at 722 K. The shoulder might be related to the H_2_O dissociation on the reduced sites of the CeZr surface.

The IR spectra show that ethanol adsorbed is promptly oxidized to acetate species reducing the catalyst. However, the acetone and CO_2_ first peaks are associated with the carboxylate species condensation, which occurs at 547 K, suggesting that the condensation is slower than the oxidation at low temperatures. At this point, the surface might be more reduced than oxidized. After that H_2_O reoxidizes the CeZr surface (H_2_ desorption at 610 K) recovering the O of the surface. At this temperature, the ethoxide species are all transformed into acetate species. Thus, the reoxidation is associated with the recovery of the basic sites. Then, the acetate species on the catalyst surface condensate again and the CO_2_ and acetone desorptions are observed at 720 K. At this point, the catalyst is reduced. Finally, H_2_O reoxidazes the CeZr surface once again (H_2_ desorption at 722 K).

The water/carboxylate species competition for the CeZr sites is depicted by the TPSR spectra. The first peak of H_2_ (610 K) occurs at a higher temperature than the ones of acetone/CO_2_ (547 K), suggesting that H_2_O is able to oxidize effectively the catalytic surface only when some carboxylate species react, i.e., acetone and CO_2_ are desorbed. The H_2 s_ peak almost occurs at the same temperature of those of acetone and CO_2_ (720 K). At this temperature, the concentration of carboxylates on the surface is lower and they do not hinder the H_2_O decomposition.

**Fig. 6:**
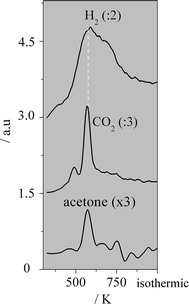
TPSR, MS spectra of CZA

Figure 6 depicts the TPSR-MS spectra of ethanol/H_2_O on CZA. Carbon dioxide, H_2_ and acetone show only one peak each at the same temperature (571 K). This result indicates that ethanol is dehydrogenated to acetaldehyde and H_2_O is decomposed on Cu^o^. Furthermore, CuO or the O adsorbed on Cu^o^ generated by the H_2_O decomposition oxidizes this aldehyde to carboxylate, and, finally, acetone and CO_2_ are synthesized. However, comparing the intensity ratios of the CO_2_ and acetone spectra, depicted on Figs. 5 and 6, it can also be suggested that acetaldehyde (see Fig. 1; Table 1) might be oxidized to acetate and, after that to CO_2_ on the Cu based catalyst.

This results show that the oxidation rate of CZA might be higher than the ketonization rate at the TPSR conditions. This might occur due to the CZA basic properties (Table 1).

The TPSR results are very different from the CZA catalytic performance depicted in Table 1 and Fig. 1. However, the experimental conditions are not the same. Since the ethanol adsorption occurs at low temperature (TPSR, see “Experimental” section), both its oxidation and the acetone synthesis are observed (Fig. 6). Indeed, a higher amount of ethanol/acetaldehyde might be adsorbed at low temperatures.

Comparing the TPSR spectra of Fig. 6 with the ones of Fig. 5, it can be observed that the former exhibits only one peak for each compound, whereas the latter two peaks. This might occur due to the lower amount of ethanol adsorbed on CZA when it is compared with CeZr, which is probably related to the lower density of acid sites of the Cu-based catalyst.

The selectivity to acetone of CZA is determined by its basicity and acidity. The former does not promote the ketonization reaction and the latter does not keep the oxygenate species adsorbed. As the acetaldehyde generation and desorption occur before the acetate condensation, the effect of the acidity is more relevant.

The mixed oxide is more active for the acetone generation when compared with ZrO_2_ not only due to its acid properties, but also due to its reducibility and reactivity towards the H_2_O dissociation. The CeZr higher concentration of vacancies promotes its reducibility [30] and the H_2_O dissociation activity. Thus, ZrO_2_ shows lower selectivity to acetone when compared with CeZr not only due to its higher acidity, but also owing to its lower vacancies concentration.

The ratios values between the acetone generation rate and the oxygenated formation rate of ZrO_2_ and CeZr are very similar (Table 1). These data demonstrate the similar behavior of the active sites of these two oxides for the acetone synthesis. One can suggest that these sites are the superficial vacancies of these oxides.

The acidity of the oxides needs to be tuned. The presence of strong acid sites should not occur as they promote the dehydration reaction. Moreover, the density of the acid sites should be high in order to keep the acetaldehyde molecules on the catalytic surface.

The redox environment of the acetone synthesis from ethanol when employing CZA, CeZr and ZrO_2_ is exhibited by the TPSR followed by the IR-MS spectroscopy.

## Conclusion

Acidity, reducibility and the H_2_O dissociation activity are very relevant catalytic properties for the acetone synthesis from ethanol. In the case of CeZr and ZrO_2_, the presence of vacancies on the oxides surface is directly associated with the reducibility and dissociation of H_2_O. The acidity of the catalysts of the acetone synthesis from ethanol needs fine-tuning in order to promote the oxygenate species adsorption and avoid the ethoxide species dehydration. The H_2_ generation during the TPSR experiments depicts the redox character of this synthesis. It was also possible to observe that CeZr is able to generate acetaldehyde, then oxidize this aldehyde to acetates species and, finally, condensate them to acetone. Moreover, CeZr dissociates H_2_O, which reoxidizes this oxide. The mechanism previously proposed by Rodrigues et al. [5] well describes the acetone synthesis when CZA, ZrO_2_ and CeZr are employed as catalysts.

## Experimental

### Catalysts

Three catalytic systems were employed: Ce_0.75_Zr_0.25_O_2_ (CeZr), monoclinic ZrO_2_ and Cu/ZnO/Al_2_O_3_ (CZA). The mixed oxide (CeZr) sample was synthesized by the coprecipitation method at conditions previously described [25]. The ZrO_2_ and CZA are commercial catalysts samples. The former was supplied by NORPRO. The composition of the latter is the following: CuO (60 wt%), ZnO (29 wt%) and Al_2_O_3_ (11 wt%). The physical mixtures were composed of CZA + ZrO_2_ and CZA + CeZr. They were gently mixed employing a small beaker and a thin glass stick. The mass ratio between the CZA catalyst and the oxides was 1.0. The particle size of the catalyst and oxides was smaller than 0.053 mm.

### Characterization

#### Infrared spectroscopy of adsorbed pyridine

Pyridine adsorption experiments followed by IR spectroscopy were carried out to probe acid properties of the catalysts using a Nicolet Magna Spectrophotometer. The spectra were recorded using thin self-supporting wafers (~20 mg). The samples were pretreated at 773 K overnight. After that the wafers were submitted to high vacuum for 2 h, then to dry oxygen pulses for 1 h and exposed again to high vacuum for 30 min at 773 K. This procedure was repeated 3 times. Pyridine was adsorbed at room temperature for 30 min at 2 Torr. The spectra were collected after desorption at 298 K for 1 h under high vacuum. The densities of the acid sites were obtained considering the absorption around 1445 cm^−1^ at 298 K and the wafers weight.

#### *Temperature*-*programmed desorption of CO*_*2*_ (TPD-CO_2_)

The experiments were carried out using a micro reactor system coupled to a QMS200 Balzers mass quadrupole spectrometer (MS). The catalysts (500 mg) were pretreated under He flow (50 mL min^−1^) at 403 K for 30 min. Next the samples were reduced at 723 K under 5% H_2_/He flow (50 mL min^−1^) for 1 h. Finally, the catalysts were oxidized at 723 K under 5% O_2_/He flow (40 mL min^−1^) for 1 h. The CO_2_ adsorption was conducted at room temperature for 1 h (25 mL min^−1^). The TPD-CO_2_ measurements were carried out heating the samples at 10 K min^−1^ up to 723 K, under He flow (50 mL min^−1^). The *m/z* = 44 fragment was continuously monitored by MS. The profiles were decomposed in Gaussian curves in order to quantify the weak, medium and strong basic sites. The basic sites, which are assigned as weak, are related to a curve which shows a maximum at a temperature lower than 443 K; the ones between 443 and 543 K, medium; and finally, the ones above 543 K, strong basic sites.

#### Temperature-programmed reduction (TPR)

The TPR analyses were performed in a multipurpose system. Initially, the catalysts were pretreated under N_2_ flow (30 mL min^−1^) at 403 K for 30 min. After that the samples were reduced at 723 K under 2% H_2_/N_2_ flow (30 mL min^−1^) for 1 h. Then, the catalysts were oxidized at 723 K under synthetic airflow (30 mL min^−1^) for 1 h. The analyses were performed using 100 mg of catalysts and 2% H_2_/N_2_ (30 mL min^−1^), from 303 to 1273 K, 10 K min^−1^, remaining at 1273 K for 30 min. The consumption of H_2_ was monitored by a thermal conductivity detector (TCD) and the TPR profiles were normalized by the samples weight and H_2_ signal intensity.

#### Temperature-programmed desorption of H_2_O (TPD-H_2_O)

The TPD-H_2_O were carried out using a micro reactor system coupled to a QMS200 Balzers mass quadrupole spectrometer. The samples (500 mg) were pretreated under He flow (50 mL min^−1^) at 403 K for 30 min. After that the catalysts were reduced at 723 K under 5% H_2_/He flow (50 mL min^−1^) for 1 h. Water vapors were generated by passing He through a saturator at 313 K. The H_2_O adsorption was conducted at room temperature for 1 h under H_2_O/He flow (50 mL min^−1^). The TPD-H_2_O measurements were carried out heating the samples at 10 K min^−1^ up to 973 K, under He flow (50 mL min^−1^). The fragments of H_2_, O_2_, H_2_O, CO and CO_2_ (*m/z* = 2, 16, 18, 28 and 44, respectively) were continuously monitored. The TPD profiles were normalized based on the N_2_ signal intensity.

#### Temperature programmed surface reaction followed by MS

*IR spectroscopies (DRIFTS)*—*In situ* IR studies were carried out using a Nicolet iS50 FT-IR spectrometer equipped with a MCT/B detector, diffuse reflectance assembly chamber (Harrick) and ZnSe window. The samples were pretreated in situ under He flow at 403 K, then they were reduced under 40% H_2_/He flow up to 723 K for 1 h. The samples were cooled again at 298 K under He flow to remove H_2_ and the corresponding background spectra were taken. Ethanol vapors were generated by passing He (20 mL min^−1^) through a saturator at 283 K. The ethanol adsorption was conducted employing an ethanol/He flow (20 mL min^−1^) for 1 h at 323 K. The TPSR desorption measures were carried out heating the sample from 323 K up to 723 K, at 20 K min^−1^ under H_2_O/He flow (40 mL min^−1^). This mixture was generated by passing He (40 mL min^−1^) through a saturator with H_2_O at 293 K. The spectra were collected at every 50 K with a spectral resolution of 4 cm^−1^ and 64 scans. The effluent was analyzed by on-line mass spectroscopy (MS). The fragments of H_2_, ethylene, CO_2_ acetaldehyde, ethanol (*m/z* = 2, 26, 44, 29 e 31, respectively) and acetone (*m/z* = 43, 58, 60) were continuously monitored.

### Catalytic tests

Catalytic tests were performed using a conventional system with a fixed bed reactor (PFR) at 1 atm and a mixture flow of 70 mL min^−1^ comprised of N_2_: H_2_O: C_2_H_5_OH = 91:8:1 mol %. Ethanol and H_2_O vapors were generated by passing N_2_ through two saturators, one at 278 K and the other at 325 K, respectively. The samples were previously dried under N_2_ flow (90 mL min^−1^) at 403 K for 30 min and reduced in situ under H_2_/N_2_ flow (10% 100 mL min^−1^) at 723 K for 1 h. The reaction rates were measured at differential conditions (conversion <10%) at 623 K. The isoconversion catalytic tests (35% conversion) were performed at 673 K and 50 mL min^−1^. The products were analyzed on-line with gas chromatograph GC Agilent 6890 equipped with two detectors (thermal conductivity and flame ionization) and a Porapak-Q/60ft column using He as the carrier gas. The samples were analyzed every 23 min during 12 h on stream. The ethanol conversion was defined as the ratio of the moles of ethanol consumed to the moles of ethanol introduced in the feed. The definition of the selectivity to one specific compound is the ratio of the number of C mol consumed to synthesize this compound to the total number of C mol consumed. The C balance was always higher than 95% for all the catalysts tested.

## Additional file

**Additional file 1: Figure S1.** Depicts the conversion of ethanol versus time on stream (TOS) at 673 K, 70 mL min^−1^, N_2_:H_2_O:C_2_H_5_OH = 91:8:1 employing CZA, ZrO_2_ and CeZr. Different masses of the catalysts were used in order to reach the isoconversion (~ 35%).

## Abbreviations

CZA: CuO/ZnO/Al_2_O_3 catalyst_
ZrO_2_: monoclinic ZrO_2 catalyst_
CeZr: Ce_0.75_Zr_0.25_O_2 catalyst_
R_oxyg_: ratio between the oxygenate compounds syntheses and ethanol total consumption rates
R_olef_: ratio between the ethylene formation and ethanol total consumption rates
R_acet_: ratio between the acetone generation and the oxygenate compounds syntheses rates
WGS: water gas shift
RWGS: reverse water gas shift
IR: infrared
FTIR: fourier transform infrared spectroscopy
DRIFTS: diffuse reflectance infrared fourier transform spectroscopy
MS: mass spectrometer
TPSR: temperature programmed surface reaction
TPD: temperature programmed desorption
TPD-CO_2_: temperature-programmed desorption of CO_2_
XRD: X-ray diffraction
TPD-H_2_O: temperature-programmed desorption of H_2_O
TPR: temperature-programmed reduction
TCD: thermal conductivity detector
PFR: fixed bed reactor
GC: gas chromatography
