# Supplementary material for: Chemicals from ethanol: the acetone synthesis from ethanol employing Ce0.75Zr0.25O2, ZrO2 and Cu/ZnO/Al2O3
Source: Chem Cent J. 2017 Apr 4;11:30. doi: 10.1186/s13065-017-0249-5 (PMC5380649; doi:10.1186/s13065-017-0249-5)
Supplement: Supplementary file 1 — Additional file 1: Figure S1. Depicts the conversion of ethanol versus time on stream (TOS) at 673 K, 70 mL min−1, N2:H2O:C2H5OH = 91:8:1 employing CZA, ZrO2 and CeZr. Different masses of the catalysts were used in order to reach the isoconversion (~ 35%). [file 13065_2017_249_MOESM1_ESM.docx]

SUPPORTING INFORMATION

Chemicals from Ethanol: the acetone synthesis From ethanol employing Ce_0.75_Zr_0.25_O_2_, ZrO_2_ and Cu/ZnO/Al_2_O_3_.

Clarissa Perdomo Rodrigues, Priscila da Costa Zonetti,

Lucia Gorenstin Appel*.

clarissa.rodrigues@int.gov.br, priscila.zonetti@int.gov.br, lucia.appel@int.gov.br

Divisão de Catálise e Processos Químicos, Instituto Nacional de Tecnologia, Av. Venezuela 82/518, CEP 21081-312, Rio de Janeiro, RJ, Brazil.

Figure S1 depicts the conversion of ethanol versus time on stream (TOS) at 673K, 70 mLmin^-1^, N_2_:H_2_O:C_2_H_5_OH=91:8:1 employing CZA, ZrO_2_ and CeZr. Different masses of the catalysts were used in order to reach the isoconversion (~35%).
